# Determination of the mass distribution of the first stars from the 21-cm signal

**DOI:** 10.1038/s41550-025-02575-x

**Published:** 2025-06-20

**Authors:** T. Gessey-Jones, N. S. Sartorio, H. T. J. Bevins, A. Fialkov, W. J. Handley, E. de Lera Acedo, G. M. Mirouh, R. G. Izzard, R. Barkana

**Affiliations:** 1 Astrophysics Group, Cavendish Laboratory, Cambridge, UK; 2 Kavli Institute for Cosmology, Cambridge, UK; 3 Sterrenkundig Observatorium, Ghent University, Krijgslaan, Belgium; 4 Institute of Astronomy, University of Cambridge, Cambridge, UK; 5 Departamento de Física Teórica y del Cosmos, Universidad de Granada, Granada, Spain; 6 Instituto de Astrofísica de Andalucía, Glorieta de la Astronomía s/n, Granada, Spain; 7 School of Physics and Mathematics, University of Surrey, Guildford, UK; 8 Department of Astrophysics, School of Physics and Astronomy, Tel-Aviv University, Tel-Aviv, Israel

**Keywords:** Stars, Early universe, Cosmology

## Abstract

The formation of the first stars and the subsequent population of X-ray binaries represents a fundamental transition in the state of the Universe as it evolves from near homogeneity to being abundant in collapsed structures such as galaxies. Due to a lack of direct observations, the properties of these stars remain highly uncertain. Here, by considering the impact of the first stars and their remnant X-ray binaries on the cosmological 21-cm signal, we demonstrate that upcoming observations have the potential to improve our understanding of these objects. We find that a 25 mK sensitivity measurement of the 21-cm global signal by a wide-beam radiometer, such as REACH, or 3,000 h of foreground avoidance observations of the 21-cm power spectrum by SKA-Low, could provide 3*σ* constraints on the mass distribution of the first stars. Such measurements will fill a critical gap in our understanding of the early Universe and aid in interpreting high-redshift galaxy observations.

## Abstract (web summary)

Next-generation radio telescopes, such as the Square Kilometre Array, will be able to reveal the mass distribution of the first stars in the Universe by detecting their impact on a faint radio signal of hydrogen atoms from Cosmic Dawn.

## Main

Observations of the cosmic microwave background (CMB) show that ~380,000 years after the Big Bang the Universe was nearly homogeneous, and visible matter was composed almost entirely of hydrogen and helium. By contrast, the Universe seen today is abundant in heavier elements, and approximately 10% of visible matter is in stars and galaxies. Building a complete picture of the transition between these disparate states is a principal focus of modern cosmology, a vital part of this picture being the first generation of stars and their compact object remnants.

The first generation of stars (Population III, Pop III) must have formed from the unenriched gas that permeated the Universe after recombination^1^. These stars produced the first heavier elements^2^ and reilluminated the Universe, thus ending the cosmic dark ages^3^ and ushering the Universe into the epoch of reionization. As these stars are composed of only hydrogen and helium, their formation mechanism, properties, evolution and ultimate fates are believed to have been distinct from the later generations of stars we observe today^1^. However, disagreement remains about the properties of the first stars, including their initial mass function (IMF, the mass distribution of stars when they reach the main sequence). For example, hydrodynamic simulations of Pop III star formation vary in their predictions for the median stellar mass by 2.5 orders of magnitude^4–9^. The uncertainty in mass distribution contributes to uncertainty in the impact of the first stars on their environment including the processes of metal enrichment^10^, reionization^11^ and heating^12^ of the high-redshift intergalactic medium (IGM), the latter principally occurring via remnant X-ray binaries (XRBs)^13^, although cosmic rays may also notably contribute to heating^14^.

Until recently, experimental probes of the Pop III IMF seemed beyond reach, with the first stars too distant and too faint to be directly observed individually^15^. Stellar archaeology provides some insights, with the null detection of Pop III stars suggesting that first-generation stars with masses <0.8 M_⊙_ (ref. ^16^) were rare. The recent potential detection of the metal signatures of a pair-instability supernova^17^ in the atmosphere of a second-generation star indicates that some Pop III stars were more massive than ~140 M_⊙_ (ref. ^18^). Furthermore, deep imaging of galaxies at *z* > 10 by the James Webb Space Telescope (JWST) potentially enables observations of a collective impact of the first stars, with the *z* = 10.6 galaxy GN-z11 garnering particular attention due to the high equivalent-width HeII emission lines from its halo^19^ possibly indicating Pop III stars with a top-heavy IMF. Intriguingly, it has also been argued that the presence of Pop III stars with a top-heavy IMF may explain the overabundance of ultraviolet (UV)-bright galaxies seen by JWST without resorting to alternatives to standard Λ Cold Dark Matter cosmology^20–22^. Measuring the Pop III IMF would thus provide insight into numerous outstanding questions in both astrophysics and cosmology.

The cosmological 21-cm signal is expected to be a uniquely powerful probe of Cosmic Dawn, revealing the typical population of high-redshift astrophysical sources inaccessible to other observables^23^. It is anticipated to provide insight into the properties of the Pop III stars, including, as we demonstrated in our previous paper^24^, the Pop III IMF. Here, we show that self-consistently including heating by Pop III XRBs strongly enhances the signature of the Pop III IMF in the 21-cm signal. We find that global signal experiments already in operation, such as the Radio Experiment for the Analysis of Cosmic Hydrogen (REACH^25^), and interferometers targeting the 21-cm power spectrum, such as the under-construction low-frequency part of the Square Kilometre Array (SKA-Low)^26,27^, will have enough sensitivity to determine the Pop III IMF, even when accounting for the uncertainties in other high-redshift astrophysics such as the properties of the metal-containing second generation of stars (Pop II) and the timing of the onset of Pop III star formation. Although previous studies have investigated the impact of Pop III high-mass XRBs on the heating and ionization of the IGM (for example, ref. ^28^), and the observable 21-cm signature (for example, refs. ^24,29,30^), this work is the first to perform a self-consistent analysis to demonstrate the promising observational prospects for the Pop III IMF.

## The Pop III IMF and the cosmological 21-cm signal

Neutral hydrogen atoms permeate the Universe between recombination and reionization. A hyperfine transition allows this hydrogen to emit or absorb 21-cm-wavelength photons from the background radiation, with the net change in the radiation referred to as the 21-cm signal (see ref. ^23^ for a review). Such a distortion produced at redshift *z* is observed at frequency *ν* = 1,420/(1 + *z*) MHz as differential brightness temperature^31^

1 $${T}_{21}(z)=\left[1-{{\mathrm{e}}}^{-{\tau }_{21}(z)}\right]\frac{\left[{T}_{{\rm{S}}}(z)-{T}_{\rm \gamma }(z)\right]}{1+z},$$

where *τ*_21_ is the local optical depth of the 21-cm line, *T*_*γ*_ is the radiation temperature of the radio background and *T*_S_ is the statistical spin temperature encapsulating the relative occupancy of the hyperfine states of neutral hydrogen. Various ongoing and under-construction experiments (for example, refs. ^25,26,32–34^) aim to measure the 21-cm signal, principally using two summary statistics: the global 21-cm signal 〈*T*_21_〉(*z*), which encodes the temporal evolution of the sky-averaged 21-cm field; and the 21-cm power spectrum Δ^2^(*k*, *z*) measuring the spatial variations in the field at wavenumber *k*.

**Fig. 1:**
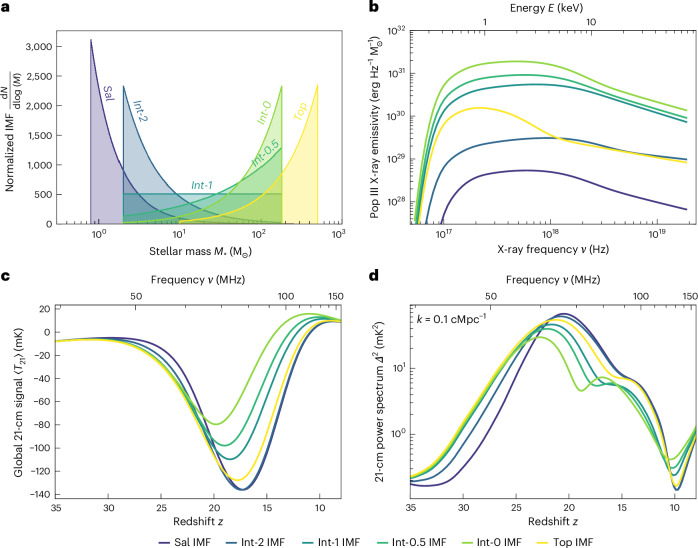
Variation of the 21-cm signal with the Pop III IMF. **a**, The six truncated power-law IMFs we consider in this work. **b**, Pop III X-ray emissivity computed for these IMFs. We observe notable variations between the emissivities, with a 257 times difference in *f*_X,III_ (integrated luminosity in the range 0.20–95.65 keV) and peak frequencies ranging from 0.92 to 3.82 keV. **c**, We find that the 21-cm global signal is sensitive to the Pop III IMF owing to its impact on the X-ray emissivity and Lyman-band emissivity of Pop III star-forming halos. **d**, A strong IMF dependence is also seen in the 21-cm power spectrum (shown at *k* = 0.1 cMpc^−1^). IMF-induced variations in X-ray emissivity are the principal cause of the large difference in the 21-cm signals at *z* < 23, while changes in the Lyman-band emissivity of stars drive the impact at *z* > 23 (Supplementary Fig. 1). To illustrate the impacts of the Pop III IMF, here we vary only the IMF while keeping other astrophysical properties fixed (see Methods for details of the simulation code and its parameterization).

The first stars are essential in shaping the 21-cm signal^3,23^. At the end of the cosmic dark ages, as the expansion of the Universe continues in the absence of stars, *T*_S_ ≈ *T*_*γ*_, and the 21-cm signal (equation (1)) nearly vanishes. However, as Pop III stars form, they emit Lyman-band radiation, enabling the Wouthuysen–Field (WF) coupling^35,36^ in the surrounding IGM and causing *T*_S_ to rapidly approach the IGM temperature. The latter is believed to be lower than *T*_*γ*_ during the dark ages and Cosmic Dawn as matter cools faster than photons in an expanding universe and astrophysical heating has yet to occur. The Pop III star formation thus leads to a rapid drop in the average 21-cm signal (equation (1) and Fig. 1) with the timing and strength of this feature sensitive to the Pop III IMF^24^. This transition is also manifested in the fluctuations of the 21-cm field as a power spectrum peak (Fig. 1).

With the build-up of the Lyman-band background produced by stars, the WF coupling causes *T*_S_ to trace the IGM temperature, thus making the 21-cm signal sensitive to any IGM heating mechanism. X-rays produced by Pop III and (later) Pop II XRBs—evolved binary systems wherein an astrophysical compact object (black hole or neutron star) is accreting matter from a donor star that is overflowing its Roche lobe—are anticipated to have been the principal cause of the IGM heating^13,14,37,38^. Stars also contribute via Ly α heating (where Ly α is a spectral line of hydrogen in the Lyman series corresponding to 1,215.67 Å); however, this channel is usually subdominant^39^. The rising IGM temperature causes the 21-cm global signal to increase and potentially switch from absorption to emission during reionization and is reflected in the 21-cm power spectrum as an inflexion point or a peak if heating is vigorous enough (Fig. 1).

The signature of the Pop III IMF in the cosmic dawn 21-cm signal was previously explored via the subdominant Ly α heating^24^ but not X-ray heating. A recent study^12^ showed that the abundance and spectra of Pop III XRBs are highly sensitive to the IMF owing to a complex interplay of factors, with the IMF impacting both the total number of Pop III binary systems and the luminosities of the individual systems. In addition, the masses of the two Pop III stars in a binary determine whether the primary star becomes a black hole or neutron star and the duration for which the secondary star undergoes Roche-lobe overflow. Hence, we anticipate X-ray heating to enhance the signature of the Pop III IMF in the 21-cm signal.

A self-consistent modelling of the Pop III IMF signature in the 21-cm signal requires all of the above mechanisms to be included. The impact of Lyman photon-mediated effects (Methods and ref. ^24^) is already included in the semi-numerical code 21CMSPACE (21-cm Semi-numerical Predictions Across Cosmic Epochs, for example, refs. ^14,37,40^). Here, we extend this code by incorporating the IMF-dependent contribution of Pop III XRBs to heating and ionization. Our X-ray spectra are calculated using state-of-the-art population synthesis simulations, which sample stellar binaries from the chosen IMF, evolve the metal-free binary systems (using BINARY_C code, for example, refs. ^41,42^) to identify systems that become XRBs, determine the evolving spectra of the XRBs using a multicolour thin disc model and apply a halo mass-dependent X-ray escape fraction. See ‘Modelling of Pop III XRBs’ in Methods and ref. ^12^ for more details on the assumptions and the uncertainties of these simulations.

We consider truncated power-law Pop III IMFs of the form

2 $$\frac{{\mathrm{d}}N}{{\mathrm{d}}M}\propto {M}^{-{\alpha }_{{\rm{III}}}},\qquad M\in [{M}_{\min },{M}_{\max }].$$

Due to the computational cost of simulating XRB catalogues, we are limited to a selection of six IMFs (Fig. 1a) with the parameters listed in Table 1. Our choices are motivated by the weak existing constraints on the Pop III IMF^1,4–9^ and include extreme IMFs dominated by low-mass (*Sal*, *α*_III_ = 2.35) and high-mass (*Top*, *α*_III_ = 0) stars. We investigate the impacts of the transition from a bottom-heavy to a top-heavy IMF (while keeping the minimum and maximum stellar mass fixed).

**Table 1:** Pop III stellar IMFs

IMF name	Colour code	IMF exponent *α*_III_	Minimum stellar mass ${M}_{\min }$ (M_⊙_)	Maximum stellar mass ${M}_{\max }$ (M_⊙_)	Relative specific X-ray emissivity *f*_X,III_
Sal (that is, Salpeter^103^)		2.35	0.8	250.0	0.29
Int-2		2.00	2.0	180.0	2.85
Int-1		1.00	2.0	180.0	35.27
Int-0.5		0.50	2.0	180.0	46.40
Int-0		0.00	2.0	180.0	75.24
Top		0.00	10.0	500.0	3.21

Each IMF is a truncated power law with minimum mass ${M}_{\min }$, maximum mass ${M}_{\max }$ and exponent *α*_III_ (see equation (2)). The Sal^103^ and Top IMFs represent intentionally extreme bottom-heavy and top-heavy scenarios, respectively, and the four Int IMFs allow us to explore the effects of varying the IMF slope while keeping the boundaries fixed. In this study, we consider Pop III stars between 0.8 M_⊙_ and 500 M_⊙_. The lower limit is motivated by stellar archaeology surveys^16^ finding that Pop III stars below this mass threshold must have been rare. The upper limit is set to 500 M_⊙_ because more massive stars experience early photodisintegration of their atmospheres, resulting in us being unable to model their spectra reliably^24^. Also given is *f*_X,III_, the computed X-ray emissivity of Pop III star-forming halos per unit star formation rate for each IMF (Methods); for convenience, these values are normalized so that *f*_X,III_ = 1 corresponds to $3\times 1{0}^{40}\,{\rm{erg}}\,{{\rm{s}}}^{-1}\,{{\rm{M}}}_{\odot }^{-1}\,\mathrm{yr}$, the specific X-ray emissivity expected for Pop II star-forming halos based on low-redshift observations and simulations^81^.

We show the specific X-ray emissivity calculated for each Pop III IMF in Fig. 1b and list the corresponding values for the integrated emissivity between 0.20 keV and 95.65 keV, *f*_X,III_, in Table 1. We find a strong dependence of specific X-ray emissivity on the IMF, with the discrepancy in *f*_X,III_ being up to a factor of 257. We find that the largest integrated X-ray luminosities occur for intermediate IMFs that strike a balance between the number of massive stars that can form a compact object and the resulting luminosity of XRBs. Bottom-heavy (*α*_III_ ≥ 2) IMFs are found to have the lowest emissivities due to a combination of having a relatively small fraction of massive stars and the resulting XRBs having less massive accretors (and, thus, lower luminosities). The extremely top-heavy Top IMF also has a moderate integrated luminosity due to fewer stars (and, hence, binaries) and shorter-lived XRBs (discussed further in Methods and ref. ^12^).

The variations in the 21-cm global signal and power spectrum caused by changing the Pop III IMF are shown in Fig. 1c and Fig. 1d, respectively. To illustrate the impacts of the IMF, we keep the other parameters of 21CMSPACE fixed to fiducial values (including cosmology and astrophysical properties such as star formation efficiencies; see Supplementary Table 1 for the complete list). As in our previous work^24^, IMF-induced differences in the 21-cm signals appear as early as *z* ≈ 30 owing to the Lyman-band effects. Because low-mass stars are less efficient Lyman-band emitters, the bottom-heavy IMFs exhibit a later onset of the 21-cm global signal absorption and a later and stronger peak in the 21-cm power spectrum at Cosmic Dawn.

Owing to the new addition of the IMF-dependent heating by the Pop III XRB population, we find that the Pop III IMF has a much stronger impact on the 21-cm signal over a wider redshift range (*z* = 10–23) than previously anticipated^24^. We find variations of up to 56 mK and Δ*z* ≈ 3 in the depth and timing of the absorption trough in the global signal, differences in the power spectrum at *k* = 0.1 cMpc^−1^ and *z* = 20 of >70 mK^2^, and an additional power spectrum peak appearing around *z* = 15 for some IMFs (for example, Int-0). These strong IMF-dependent signatures are predominantly caused by the difference in *f*_X,III_, with the spectral shape variations having a subdominant impact (Supplementary Fig. 1). This also explains why the three IMFs with lower emissivities (Sal, Int-2 and Top) have similar 21-cm signals, as, in these cases, heating is dominated by the Pop II XRBs.

The details and the strength of the IMF-driven variations in the 21-cm signal will depend on other astrophysical processes, which remain uncertain. For example, cosmic ray heating^14^ from Pop III supernovae may be efficient and enhance the signature of the Pop III IMF due to intrinsic links between the rate and types of supernovae and the IMF. Despite these uncertainties, the above examples are sufficient to demonstrate that the Pop III IMF can strongly impact the 21-cm signal. This finding has two major consequences for 21-cm cosmology. First, measurements of the 21-cm signal can potentially be used to constrain the IMF of the first generation of stars. Second, assuming an incorrect IMF model could bias the inference of other astrophysical and cosmological information from the 21-cm signal (for example, the nature of dark matter^43^). A realistic assessment of the significance of these constraints and estimates of biases requires specifying expected experimental sensitivities, which we will address next.

## Synthetic 21-cm measurements and the Pop III IMF inference

The field of observational 21-cm cosmology is rapidly evolving, with ongoing experiments (for example, refs. ^33,34,44–47^) progressively improving upper limits and the under-construction SKA scheduled for commissioning in 2028. Here, we consider prospective constraints on the Pop III IMF using the sensitivities of the operational REACH global signal experiment and the SKA as examples.

REACH^25^ is a state-of-the-art experiment to detect the 21-cm global signal using multiple radiometers and a comprehensive Bayesian analysis pipeline^48^. At the time of writing, REACH is undertaking its first phase of observations. In their mission paper^25^, the REACH collaboration outlines three potential scenarios for the noise-limited (a white Gaussian noise model is used) post-foreground removal global 21-cm signal measurement with sensitivities of 250 mK (pessimistic), 25 mK (anticipated) or 5 mK (optimistic) at a 0.1 MHz resolution across the redshift band of *z* = 7.5–28.0. This is equivalent to 79.1, 7.9 and 1.6 mK sensitivity, respectively, when integrated over 1 MHz bins. In our subsequent analysis, we consider the same three sensitivity cases and adopt the same assumptions. Our REACH forecasts represent a best-case scenario where foreground removal does not produce a strong systematic uncertainty in the measured signal^49^.

The principal goal of SKA-Low, the low-frequency radio interferometer part of the SKA observatory^26^, is to detect the 21-cm power spectrum over the *z* = 7.0–27.0 redshift range. To model future measurements by this experiment, we use existing sensitivity forecast for 300, 1,000 and 3,000 h of observations by the SKA-Low core^27^ and assume achromatic Gaussian noise. We simulate a foreground avoidance strategy by considering measurements only at *k* > 0.1 cMpc^−1^ and are limited to *k* ≤ 1.0 cMpc^−1^ by the SKA-Low core resolution. Our forecast for the SKA is conservative and should be treated as a worst-case scenario, as we consider only the spherical 21-cm power spectrum measurement by the SKA-Low core in a limited wavenumber range. The full experiment is expected to perform better due to the additional information from the cylindrical 21-cm power spectrum^50^, foreground mitigation and access to higher wavenumbers.

At this juncture, we should discuss the disputed detection of the 21-cm global signal by the Experiment to Detect the Global EoR Signature (EDGES) low-band instrument^32,51,52^. If the detection is of cosmological origin, its anomalous depth would suggest exotic physics that enhances the 21-cm signal, such as dark matter cooling (for example, ref. ^43^) or a strong radio background in addition to the CMB (for example, ref. ^53^). Any physical processes that enhance the 21-cm signal would also increase the differences between models with different Pop III IMFs, reducing the sensitivity required for constraints. However, the EDGES detection may not be cosmological^51,52^, and, thus, we do not attempt to use the EDGES data in this study. Furthermore, to ensure a conservative assessment of the Pop III IMF constraints, we include only a weak astrophysical radio background that is negligible compared with the CMB (<2.8% at all redshifts^54^) and do not include dark matter cooling when simulating the 21-cm signal. We also do not consider the existing bounds on the 21-cm global signal from SARAS 3 (a spectral radiometer for probing Cosmic Dawn and the Epoch of Reionization)^33^ or the power spectrum limits from Hydrogen Epoch of Reionization Array (HERA)^55^ as they only slightly disfavour high Pop III star formation efficiencies^56^ and so are not yet sufficiently constraining to provide insight into the IMF.

To make our forecasts as realistic as possible, we generate synthetic experimental measurements, which combine 21-cm signals created using 21CMSPACE and additive noise designed at the right level and form to match each experiment and sensitivity. We adopt a nested-sampling-based Bayesian methodology for the analysis of this data, inspired by the approach taken by REACH^25^. This methodology allows us to rigorously marginalize over uncertain high-redshift astrophysical parameters such as the Pop III star formation efficiency and properties of Pop II stars and XRBs (see Supplementary Table 1 for a full list of the parameters we fit for and their intentionally broad priors). Such an approach is essential to guarantee our forecasts properly account for any degeneracies between the impacts of the Pop III IMF and those of other astrophysics on the 21-cm signal (discussed further in ‘Simulations of the 21-cm signal’ section in Methods).

As a single nested sampling run would require millions of 21-cm signal simulations, 21CMSPACE is too slow for direct use in this analysis owing to its approximately 3-h runtime. To remedy this issue, we create neural network emulators of 21CMSPACE and use these in our likelihoods and synthetic data generation, following the methodology applied to the analysis of the existing HERA and SARAS 3 data in our earlier work (for example, ref. ^57^) (see Methods for more details).

## Results

Although the cosmic Pop III IMF is highly uncertain, we must assume an IMF to generate synthetic measurements. We refer to this as the data IMF, and it serves as the ground truth in simulation and data analysis. Unless otherwise stated, we adopt Int-1 as the data IMF, as it is consistent with current theoretical expectations^1^.

**Fig. 2:**
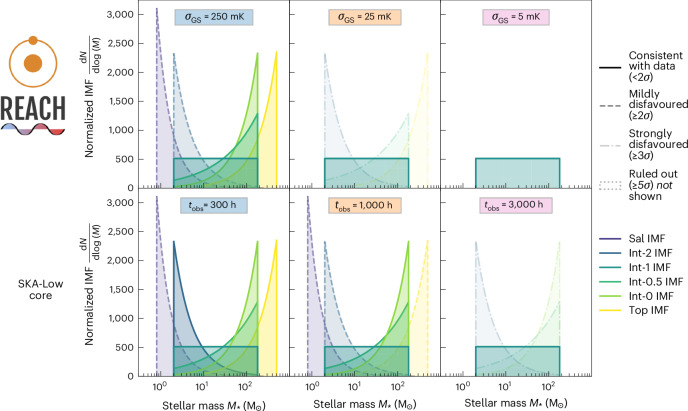
Prospective constraints on the mass distribution of the first stars from 21-cm signal experiments. For this figure, the synthetic measurement data were generated using the Int-1 IMF indicated in bold in the legend. Each panel shows the six Pop III IMFs we consider in this study (Fig. 1 and Table 1), with the marginalized a posteriori confidence in each IMF indicated via its line type and opacity. The top row shows prospective IMF constraints from the 21-cm global signal experiment REACH at 250, 25 and 5 mK sensitivity (from left to right), and the bottom row shows constraints from the 21-cm power spectrum experiment SKA-Low with 300, 1,000 and 3,000 h of observations (also from left to right). REACH at 25 mK and SKA-Low at 3,000 h are both found able to identify the correct IMF (Int-1) at >3*σ* confidence, showing the potential of the 21-cm signal to determine the Pop III IMF.

We depict the prospective constraints (posterior distributions) on the Pop III IMF in Fig. 2 for REACH and SKA-Low at different sensitivities. In all cases, we find that the data IMF (Int-1) is always correctly identified as being consistent with the data, and the five alternative IMFs (for example, non-data IMFs) are disfavoured at increasing statistical significance as sensitivity improves. For REACH at 250 mK sensitivity, we find only mild disfavouring (≥2*σ*, where >*Xσ* indicates that the probability of an event occurring is less than that of a normally distributed variable deviating from its mean by more than *X* standard deviations (*σ*)) of the two IMFs that have the weakest Pop III contribution to X-ray heating (Sal and Int-2 IMF) and, thus, produce the strongest 21-cm signals that are most readily excluded by the data. At 25 mK, all the alternative IMFs are disfavoured at >3*σ*, with the significance increasing to >5*σ* at 5 mK. Our conservative SKA-Low forecast is still very promising. We find that with 300 h of observations SKA-Low would only mildly disfavour one alternative IMF (Sal), while 1,000 h of observations will allow us to disfavour three alternative IMFs. With 3,000 h, we can detect the correct data IMF at >3*σ* with some alternative IMFs ruled out at much higher significance (>5*σ*). To test the robustness of our findings, we repeat the procedure five more times, rerunning the analysis with each of our six IMFs as the data IMF. We find that the data IMF is always consistent with the synthetic measurement, and REACH at 25 mK sensitivity, or 3,000 h of integration with SKA-Low, is sufficient to constrain the IMF at >3*σ*. The results are shown in Supplementary Figs. 2–4.

In addition to the forecasts for individual experiments, we considered a joint analysis between REACH at 25 mK sensitivity and 1,000 h of SKA-Low observations (see Supplementary Fig. 2 for other combinations). As expected, the joint analysis improves the IMF constraints, with a >6-fold decrease in the posterior probability of all alternative IMFs, thus motivating future synergies to increase the statistical significance of the Pop III IMF constraints.

Our methodology also allows us to demonstrate the biases introduced by assuming an incorrect Pop III IMF when analysing the 21-cm data (Supplementary Fig. 5). We find that the impacts of the Pop III IMF on the 21-cm signal can be partially compensated for by varying other cosmic dawn and reionization astrophysical parameters, resulting in biased inference of these other parameters if an incorrect IMF is assumed. These biases are found to increase with experimental sensitivity; for example, in a 25 mK global 21-cm measurement, we see >2*σ* biases in some Pop II properties, which rises to >5*σ* biases in a 5 mK measurement. It will thus be necessary to consider uncertainties in the Pop III IMF to ensure accurate interpretation of future precision 21-cm signal measurements.

## Discussion and conclusions

We explored the sensitivity of the 21-cm signal to the Pop III IMF, finding that it was substantially underestimated by previous analyses^24^, which neglected the impact of the IMF via Pop III XRB heating. We predict that 21-cm signal experiments should reveal the mass distribution of these elusive first stars, finding that >3*σ* significance constraints on the Pop III IMF can be achieved either by a noise-limited measurement of the 21-cm global signal at 25 mK sensitivity (for example, with REACH) or by 3,000 h of SKA-Low 21-cm power spectrum observations. A joint analysis of these two observables can further increase the significance.

Our conclusions are found to be robust to the uncertainty in Pop III IMF and were derived using a nested-sampling Bayesian methodology to account for the potential degeneracies with other poorly constrained high-redshift astrophysics such as Pop III and Pop II star formation efficiencies. Although we do not include all possible stellar mass-dependent effects (for example, cosmic ray heating and gradual metal enrichment are omitted for simplicity), their self-consistent modelling is expected to further enhance the dependence of the 21-cm signal on the Pop III IMF, thus strengthening our conclusions. Furthermore, while there remain uncertainties in Pop III XRB formation, we anticipate these either to be IMF independent or to have a relatively weak impact on the IGM heating and the 21-cm signal, thus having a subdominant effect on the predicted constraints.

A noise-limited measurement of the global 21-cm signal at 25 mK may prove difficult due to the challenge of removing the strong radio foregrounds. However, our SKA-Low forecasts are intentionally conservative. They use a foreground avoidance strategy, consider only the array core and use the spherical 21-cm power spectrum instead of the cylindrical one. Consequently, the full SKA-Low is expected to achieve stronger IMF constraints in less observation time than our estimates above. With SKA-Low commissioning expected in 2028 and other experiments in progress attempting to make conclusive global signal (for example, refs. ^33,44^) or power spectrum detections (for example, refs. ^34,45–47^), the prospect for 21-cm cosmology to measure the masses of the first stars in the coming years is exciting.

The insights gained from measuring the Pop III IMF via the 21-cm signal will shed light on cosmic dawn, a critical and largely uncertain transition in cosmological history. Furthermore, this measurement will elucidate high-redshift galaxy measurements by independently testing the ‘top-heavy Pop III’ explanation of certain JWST observations^19,21,22^. It will also synergize with the next-generation gravitational wave observations of black hole–black hole mergers at *z* > 10 (ref. ^58^). Thus, our findings provide an additional compelling case for 21-cm cosmology.

## Methods

### Modelling of Pop III stellar spectra

In our 21-cm signal simulations, we utilize the Pop III star spectra computed for our previous paper^24^. Here, we summarize the methodology of that work for completeness (see the original paper for further details and a discussion of the spectra).

The Pop III population spectra were calculated by combining stellar evolution tracks with a grid of precomputed stellar spectra. These stellar evolution tracks were simulated^42^ using the Modules for Experiments in Stellar Astrophysics (MESA) stellar evolution code^59–63^ (version 12115), assuming the stars evolved in isolation (discussed further at the end of this section), were non-rotating, were initially metal-free (that is, had metallicity *Z* = 0) and had negligible mass loss. The tracks begin at the zero-age main sequence and end at core hydrogen depletion (for stars of masses *M* < 310 M_⊙_) or photo-evaporation (stellar masses *M* > 310 M_⊙_). Meanwhile, the stellar atmosphere modelling code TLUSTY^64–67^ (version 205) was utilized to calculate stellar spectra on a grid of effective temperature and surface gravity values. This grid was designed to cover the aforementioned stellar evolution tracks. Atmospheres were calculated using non-local thermodynamic equilibrium modelling with atomic and ionic lines included^68^. Chemically, the atmospheres were modelled as being composed of only hydrogen and helium in Big Bang nucleosynthesis proportions. This is motivated by the expectation that Pop III stars formed metal-free and by our stellar evolution simulations predicting that negligible amounts of metals synthesized within the stars reach their surface during their main sequence.

The lifetime spectra of individual Pop III stars were then calculated by integrating along each stellar evolution track, interpolating the grid of stellar spectra to each point on the evolution track. These individual stellar spectra were subsequently incorporated into 21CMSPACE (see ‘Simulations of the 21-cm signal’ section), with the Pop III population-averaged Lyman-band (and Lyman–Werner band) emissivity finally computed at runtime by integrating these spectra weighted appropriately by the Pop III IMF.

A recent study^69^ found that binary evolution and mass transfer increase the total UV emission of Pop III binaries when compared with the same stars evolving in isolation. By combining their worst-case results (largest difference) with predictions for the Pop III interacting binary fraction^4,69^, we can estimate that neglecting these mechanisms in 21CMSPACE has led to an underestimation of the Pop III Lyman–Werner emissivities by, at most, 25%. Hence, while not modelling a portion of Pop III stars as binaries when calculating Lyman-band emissivities is somewhat inconsistent with the XRB modelling of the next section, the impact of this assumption is relatively small.

### Modelling of Pop III XRBs

Our modelling of Pop III XRBs follows a two-step methodology introduced in ref. ^12^. In the first step, we create catalogues of metal-free (*Z* = 0) binaries at high redshifts for all six explored IMFs. To do this, we take 21CMSPACE simulations of the Pop III star formation rate and sample them according to the IMF to create a catalogue of stars forming at each redshift. We then compute the number of newly formed binary systems by assuming 28% of Pop III stars are in binaries (note that other works sometimes instead quote the fraction of stellar systems that are binaries). The utilized value for binary fraction is intentionally conservative, being approximately half the value found in some of the hydrodynamic simulations of Pop III protostar multiplicity^4,70,71^. As we do not use a mass-dependent pairing of stars in a binary system, any error in the assumed binary fraction should scale the X-ray emissivities of each IMF by the same factor. Thus, the current uncertainty in Pop III binary fractions introduces at most a factor-of-3 variation in the predicted Pop III X-ray emissivity. Consequently, this uncertainty does not strongly impact our forecast constraints as we find the variation in the total Pop III X-ray emissivity between IMFs to be ${\mathcal{O}}(300)$ (Table 1). With the number of binaries established, we assign a subset of the sampled stars to those systems, pairing them randomly. Then, we set the initial orbital parameters for each binary system by Monte-Carlo sampling of mass-dependent observationally motivated probability distribution functions^72–74^.

In the second step, the catalogue of binaries is fed as an initial condition to the population synthesis code BINARY_C^41,42,75–78^. For self-consistency in our BINARY_C simulations, we use the same metal-free stellar evolution tracks as we used to compute the Pop III stellar spectra (see previous section). A binary is classified as an XRB whenever the primary is a black hole or neutron star and is accreting material from a companion via winds or Roche-lobe overflow. We then calculate the XRB’s spectral energy distribution (SED), assuming a thin accretion disk model, taking into account Comptonization by a corona, the accretion rate, the mass of the primary compact object and the binary’s orbital parameters. We model the X-ray escape fraction from the host halo (following ref. ^12^) by assuming halo-mass dependent absorption and adopting primordial gas composition (76% of atomic hydrogen and 24% of helium). The X-ray emission of each XRB is assembled by BINARY_C into a catalogue of XRB lifetimes and energy emission across the frequency range 4 × 10^−8^ to 4 × 10^10^ keV. In addition, we compute the local absorption of X-rays by hydrogen and helium within dark matter halos where the XRBs are situated to obtain the X-ray spectrum that would escape and affect the IGM.

Finally, we normalize the total escaping X-ray emissivity using the simulated star formation rate density to find the specific X-ray emissivity from Pop III star-forming halos. These population-averaged emission spectra (shown in Fig. 1) were then integrated into 21CMSPACE as described in the next section.

For 21-cm cosmology, the relevant part of the X-ray spectrum is from 0.1 to 2.5 keV. X-rays at these energies can readily escape into the IGM^12^ but are still absorbed in a Hubble time^23^ and, thus, contribute to heating the IGM. We find a large portion of the X-ray emission of Pop III XRBs lies in this range, with the peak of the X-ray emissivities either lying within this band (0.9, 2.0, 2.4 and 2.4 keV for the Top, Int-0, Int-0.5 and Sal IMFs, respectively) or just above it (3.0 keV for the Int-1 IMF and 3.8 keV for the Int-2 IMF). Hence, based on the spectra alone, we anticipate that Pop III XRBs could efficiently contribute to the X-ray heating of the IGM (in agreement with literature, for example, ref. ^28^) with the signature of this heating being IMF dependent.

Alongside these variations in peak frequency, we observe that the integrated specific X-ray emissivity *f*_X,III_ (Table 1) differs by a factor of 257 between IMFs (as highlighted in the main text). The sensitivity of X-ray emissivity to the Pop III IMF stems from the interplay of four key mechanisms^12^: the number of Pop III stellar binaries that form, the proportion of binaries that become XRBs, the lifetime of the XRBs and the spectra of the individual XRBs. Bottom-heavy IMFs lead to more Pop III stars and stellar binaries; however, these stars are generally of lower mass, and thus few of these binaries have primaries large enough to become the neutron stars or black holes required to form an XRB. Hence, the proportion of binaries that become XRBs tends to increase as the IMF becomes more top-heavy (this is somewhat complicated by pair-instability supernovae causing some massive Pop III stars to leave no remnant behind^17^). In addition, the typical primary star mass increases as the IMF becomes more top-heavy, leading to a greater accretion rate and more efficient X-ray emission, producing individual XRBs that are more luminous on average. However, the typical XRB lifetime simultaneously decreases due to the increasing mass of the secondary star. Thus, we see these four competing effects, with the number of binaries forming and XRB lifetime being highest for bottom-heavy IMFs and the proportion of binaries that become XRBs and XRB luminosity being highest for top-heavy IMFs. The balance of these mechanisms leads to the trends seen in Fig. 1 and Table 1, where the intermediate IMFs have the highest X-ray emissivities, with extremely top-heavy or bottom-heavy IMFs having lower emissivities. A more detailed discussion of these effects and their impacts on the X-ray emission properties of Pop III star-forming halos can be found in ref. ^12^.

Some aspects of Pop III XRB formation and evolution, such as supernova kicks, common-envelope evolution and mass accretion, are poorly understood, and, thus could present an obstacle to definitively determining the Pop III IMF from the 21-cm signal. Robustly assessing the impact of each of these uncertain processes demands quantifying their effects on the 21-cm signal and propagating this through a forecasting analysis, which is beyond the capabilities of state-of-the-art simulations and the scope of this Article. However, we argue that the uncertainties mentioned above are expected to have a smaller impact on the observable signal than the Pop III IMF. Thus, while they may somewhat weaken our constraints on the Pop III IMF, they are unlikely to eliminate them. First, we have used BINARY_C to test several supernova kick distributions based on observational data of pulsar velocities^79,80^ finding that the impact on the number of XRBs is small, being at most 10%. Second, the effect of differences in common-envelope evolution on Pop II XRBs has previously been explored in the literature with a strong effect reported on low-mass XRBs; by contrast, high-mass XRBs were found to be largely unaffected^81^. Although an analogous study of Pop III XRBs does not exist, we expect a low impact on our results as the majority of Pop III XRB X-ray emission in our work is due to high-mass XRBs. Finally, as Pop III XRBs are overwhelmingly powered by Roche-lobe overflow (the absence of metals makes line-driven winds very inefficient), changes in mass transfer efficiency scale the average luminosity of Pop III XRBs in an IMF-invariant manner. Hence, like the binary fraction, this introduces an IMF-independent scaling factor in the X-ray luminosity that is probably much smaller than the ${\mathcal{O}}(300)$ variation between IMFs.

### Simulations of the 21-cm signal

To simulate the 21-cm signal, we utilize 21CMSPACE^14,24,37,39,40,54,82–88^. The core idea behind the code is a separation of scales between large-scale phenomena, such as radiative transfer and the variations in the overdensity of the Universe, and small-scale phenomena, such as dark-matter halo collapse and star formation. These small-scale phenomena are not resolved in the simulations; instead, they are modelled using analytic approximations or fitting formulas of finer-resolution simulations. Conversely, large-scale phenomena are resolved, with the simulation volume split into a grid of 128^3^ cells of side length 3 cMpc. Such large cells are necessary for three main reasons: to ensure cells remain in the linear growth regime throughout the simulation, to give sufficient cell volume for some of the aforementioned analytic models to be valid and to allow for simulation volumes large enough for accurate statistics on the cosmological observables of interest while keeping the simulation runtime reasonably short.

21CMSPACE models a wide range of physics known to impact the 21-cm signal including, but not limited to: Pop III and Pop II star formation and radiative emission^24,88^, the WF coupling^35,36^, Lyman–Werner feedback^83^, baryon-dark matter relative velocity feedback^82^, X-ray heating and ionization^37^, Ly α heating^39^, CMB heating^85,89^, Ly α multiple scattering^39^, photoheating feedback^84^, reionization^37^, the suppression of star formation efficiencies in low-mass halos^83^ and redshift-space distortions^90^. Detailed descriptions of how 21CMSPACE implements these physical processes and a comprehensive list of its features can be found in the most recent code development papers^14,87,91^. Here, we limit our discussion to the aspects of the code modified as part of this study to incorporate the Pop III IMF dependence of X-ray heating via the properties of Pop III XRBs.

In our updated version of 21CMSPACE, we add the capability of setting separate Pop II and Pop III specific X-ray emissivities and SEDs (previously, they were assumed to be the same). Specifically, each star-forming halo is modelled as emitting X-rays in the energy range 0.20–95.65 keV with a luminosity ${L}_{{\rm{X}}}^{{\rm{halo}}}$ in proportion to its star formation rate SFR^halo^ (for example, ref. ^23^)

3 $$\frac{{L}_{{\rm{X}}}^{{\rm{halo}}}}{{{\rm{SFR}}}^{{\rm{halo}}}}=\left(3\times 1{0}^{40}{f}_{{\rm{X}},{\rm{j}}}\right)\,{\rm{erg}}\,{{\rm{s}}}^{-1}\,{{\rm{M}}}_{\odot }^{-1}\,{\rm{yr}}\quad j\in \{{\rm{II}},{\rm{III}}\},$$

where the proportionality constant *f*_X,*j*_ is the integrated specific X-ray emissivity of the halo, which is now set separately for Pop II and Pop III star-forming halos as *f*_X,II_ and *f*_X,III_ respectively. We normalize the proportionality constants in equation (3) to $3\times 1{0}^{40}\,{\rm{erg}}\,{{\rm{s}}}^{-1}\,{{\rm{M}}}_{\odot }^{-1}\,{\rm{yr}}$, as this is the theoretically and observationally predicted value for Pop II star XRBs^13,81^, that is, in our notation these studies predict *f*_X,II_ = 1, although with a non-negligible uncertainty (see below). Although equation (3) was originally proposed for Pop II stars, it has also been found in simulation to hold well for Pop III stars across a range of IMFs^12^ (the constant *f*_X,III_ being IMF dependent), motivating our usage of it for both stellar populations. The total luminosity derived from equation (3) is then assumed to be distributed across frequency according to the relevant SED, SED_II_ for Pop II star-forming halos (in this work, we adopt the Pop II SED from ref. ^13^) and SED_III_ for Pop III star-forming halos (see previous section). In 21CMSPACE, the distribution of Pop II and Pop III star-forming halos within each simulation cell is modelled statistically using an analytic star formation prescription^88^. Hence, the X-ray emissivity of each cell is, in practice, calculated from the cell’s Pop II and Pop III star formation rate rather than those of individual halos. Finally, we modified 21CMSPACE to automatically assign *f*_X,III_ and SED_III_ according to the Pop III IMF and the dataset of specific X-ray emissivities calculated in the previous section (for example, the *f*_X,III_ values listed in Table 1). As a result, both Pop III X-ray and Lyman-band emissivity are now consistently modelled from the Pop III IMF in 21CMSPACE, as the latter was introduced into the code in our previous study^24^.

In addition to the Pop III IMF, 21CMSPACE takes various high-redshift astrophysical parameters as free inputs because their values remain uncertain and they are independent of the Pop III IMF. These parameters are described in Supplementary Table 1, alongside the broad priors we use for each parameter during our Bayesian analysis to encompass the range of current theoretical uncertainty in their values. In particular, note that, while we derive *f*_X,III_ and SED_III_ from the Pop III IMF, *f*_X,II_ and SED_II_ are still free independent input parameters to the code. For our forecasts, we treat *f*_X,II_ as an unknown to be fit for alongside the Pop III IMF while fixing SED_II_ to a theoretically predicted spectrum^13^.

Because *f*_X,II_ and the Pop III IMF both impact X-ray heating, varying them has a similar, but not identical, impact on the 21-cm signal. Consequently, a change in one of these variables can be partially compensated for by a change in the other, leaving the predicted signal only marginally altered. As a result, if, like in this work, both *f*_X,II_ and the Pop III IMF are fit simultaneously, the posteriors on the potential values of these parameters are correlated, that is, knowledge of one conveys a degree of knowledge of the other. If we were instead to fix *f*_X,II_ to the data generation value, we would be implicitly leveraging additional information unavailable to us for a real 21-cm measurement. This would lead to spuriously tighter constraints on the predicted Pop III IMF. It is thus essential, for the sake of the realism of this exercise, that we simultaneously fit for any parameters of 21CMSPACE, like *f*_X,II_, whose impact on the 21-cm signal overlaps with that of the IMF. An additional consequence of this compensation between *f*_X,II_ and the Pop III IMF is that, if the Pop III IMF is fixed to an incorrect value, the inferred value of *f*_X,II_ would be biased or vice versa (Supplementary Fig. 5). Hence, in the case of actual data wherein the correct *f*_X,II_ would be unknown, fixing its value would not only lead to erroneously tight constraints but probably bias them as well.

In this study, we utilize the Δ^2^(*k*, *z*) 21-cm power spectrum convention

4 $${\varDelta }^{2}(k,z)=\frac{{k}^{3}}{2{\uppi }^{2}}{P}_{{\rm{21}}}(k,z),$$

with

5 $$\left\langle {\tilde{T}}_{{\rm{21}}}\left({\bf{k}},z\right){\tilde{T}}_{{\rm{21}}}^{* }\left({{\bf{k}}}^{{\prime} },z\right)\right\rangle ={(2\uppi )}^{3}{\delta }^{3}\left({\bf{k}}-{{\bf{k}}}^{{\prime} }\right){P}_{{\rm{21}}}(k,z),$$

where ${\tilde{T}}_{{\rm{21}}}$ is the Fourier transform of the 21-cm brightness temperature field, *δ*^3^ is the three-dimensional Dirac delta function and 〈〉 is the ensemble average.

### Emulation of the 21-cm signal

As discussed in the main text (and in ‘Nested sampling and Bayesian analysis methodology’ section), we analyse synthetic 21-cm signal measurements using a nested-sampling-based methodology to ensure reliable forecasts. A nested sampling analysis typically requires millions of likelihood evaluations and, therefore, simulations of observables. Direct use of 21CMSPACE within such an analysis is computationally unfeasible due to its runtime on the order of hours. Hence, we create neural network emulators of 21CMSPACE, following existing 21-cm data analyses (for example, ref. ^57^). These emulators provide a six-order-of-magnitude speed-up, making a nested sampling analysis feasible. For ease of quantification of emulation error and comparison with previous works, we train a separate emulator for the 21-cm global signal and 21-cm power spectrum for each of our six Pop III IMFs (12 emulators total).

Our 12 neural network emulators of 21CMSPACE are based on those used in previous 21-cm data analyses^34,55,57,92^, with GLOBALEMU^93^ used for the 21-cm global signal emulators, and the SCIKIT-LEARN^94^ multilayer perceptron used for the 21-cm power spectrum emulators. Each global signal emulator has 5 hidden layers of 16 nodes, and the power spectrum emulators have 4 hidden layers of 100 nodes. Both network architectures follow a GLOBALEMU-like methodology of taking the redshift, and in the case of the power spectrum emulators the wavenumber, as inputs to ensure the output 21-cm signals are smooth^93^. To train the networks, we ran 10,000 21CMSPACE simulations for each Pop III IMF (60,000 simulations total), randomly sampling the input astrophysical parameters from their priors listed in Supplementary Table 1. The 10,000 simulation outputs for each IMF, covering 7 ≤ *z* ≤ 39 and 0.085 ≤ *k* ≤ 1 cMpc^−1^, were then split using a test-to-train ratio of 0.1 to form the testing and training set of the global signal and power spectrum emulator for that Pop III IMF.

So that we can compare the accuracy of our emulators to previous works^55,92^, we utilize the same error metrics as those studies: the root-mean-square error for the 21-cm global signal emulators, and the root-mean-square of the error metric

6 $${\varepsilon }_{{\rm{power}}}(k,z)=\frac{{\varDelta }_{21{\rm{CMSPACE}}}^{2}(k,z)-{\varDelta }_{{\rm{emulator}}}^{2}(k,z)}{{\varDelta }_{21{\rm{CMSPACE}}}^{2}(k,z)+0.1\,{{\rm{mK}}}^{2}},$$

for the 21-cm power spectrum emulators. The latter, which we shall refer to as the root-mean-square modified fractional error, was introduced due to the large dynamic range of the 21-cm power spectrum and to improve emulator training by down-weighting errors when the 21-cm power spectrum is too small to be measured. Overall, our emulator errors are comparable to those of the aforementioned previous studies, with a mean root-mean-square error <10 mK for all global 21-cm signal emulators, and a mean root-mean-square modified fractional error <8% for all 21-cm power spectrum emulators. A full breakdown of the performance metrics computed for our 12 emulators is given in Supplementary Table 2.

### Synthetic measurement data generation

We combine a predicted 21-cm signal and a noise realization to form our synthetic measurement datasets. The former is generated by evaluating the emulator corresponding to the chosen data IMF and observable of interest at the synthetic data parameter values listed in Supplementary Table 1. Where possible, these values are chosen to be consistent with theoretical or observational expectations to ensure our forecasts are as realistic as possible within current uncertainties. The noise is specific to each experiment, its configuration and the observation time or sensitivity.

For REACH, we assume an observation band covering frequencies 49.0–167.1 MHz at 0.1 MHz resolution in agreement with the mission paper^25^. Furthermore, we assume the noise on the measured signal is a white Gaussian noise with a standard deviation of 250, 25 or 5 mK for the respective sensitivity. Combining a realization of this white noise with a 21-cm global signal at the REACH resolution gives us our synthetic REACH observations.

Similarly, for SKA-Low, we follow preexisting noise estimates^27^ for 1,000 h of observations, scaling to the 300 and 3,000 h equivalents, using the fact that power spectrum sensitivity goes as the reciprocal of integration time. These noise estimates assume observations by the SKA-Low core across 50.7–177.5 MHz in 1.0 MHz frequency bins, and that wavenumbers are integrated into 1 dex bins, with UV plane coverage of the array, the array filling factor and thermal noise all taken into account. While the SKA-Low noise estimates and baselines cover a wider wavenumber range, in this study, we limit our synthetic observations to 0.1 ≤ *k* ≤ 1.0 cMpc^−1^. The lower limit is used to mimic a foreground avoidance strategy (see ref. ^95^, for a discussion of foreground avoidance versus foreground mitigation), and the upper limit is a practical limitation set by the resolution of the SKA-Low core. The resulting noise levels vary strongly with wavenumber and redshift. Hence, we model SKA-Low noise as achromatic Gaussian noise, adding it to 21-cm power spectrum predictions at the same frequency and wavenumber resolution to form our synthetic SKA-Low datasets.

### Nested sampling and Bayesian analysis methodology

We perform a nested-sampling-based Bayesian analysis^96^ on each of our synthetic measurement datasets using POLYCHORD^97,98^. We choose to use this analysis paradigm owing to its natural ability to account for parameter degeneracies, and because it is the approach taken by REACH and some existing 21-cm data analysis (for example, refs. ^55,56,92^). In a Bayesian analysis, the prior *π*(*θ*), our initial knowledge of the parameter values, is revised in light of some data *D* through the application of Bayes theorem

7 $${\mathcal{P}}(\theta | D)=\frac{{\mathcal{L}}(D| \theta )\pi (\theta )}{{\mathcal{Z}}},$$

into our updated knowledge of the parameter values, ${\mathcal{P}}(\theta | D)$, the posterior. Above ${\mathcal{L}}(D| \theta )$ is the likelihood (for example, probability) of seeing the data given a set of parameter values, and ${\mathcal{Z}}$ is the Bayesian evidence, a measure of the goodness of fit of the underlying model to the data given by

8 $${\mathcal{Z}}=\int{\mathcal{L}}(D| \theta )\pi (\theta ){\mathrm{d}}\theta .$$

Constraints on the parameters can be inferred from ${\mathcal{P}}(\theta | D)$ (for example, Supplementary Fig. 5) and alternative data models compared via ${\mathcal{Z}}$ (for example, Fig. 2).

For our analysis, we treat the six Pop III IMFs as alternative models rather than as a parameter. Consequently, we perform six nested sampling analyses for each of our synthetic measurement datasets, each with a fixed IMF and producing a separate posterior and Bayesian evidence ${{\mathcal{Z}}}_{i}$. This method allows us to investigate both the biases caused by the assumption of an incorrect IMF (from the output posteriors) and the prospective constraints on the Pop III IMF (from the output evidence) using the same nested sampling runs. Formally, the marginalized posterior probability on each IMF is given by

9 $${\mathcal{P}}({\rm{IMF}}| D)=\frac{{{\mathcal{Z}}}_{{\rm{IMF}}}}{\mathop{\sum }\nolimits_{i}^{{\rm{IMFs}}}{{\mathcal{Z}}}_{i}},$$

where the sum is over all six considered IMFs, and in using this formula, we are implicitly assuming a discrete uniform prior over the IMFs.

In our analysis, we thus use a discrete uniform prior over IMFs and the priors specified in Supplementary Table 1 for the other uncertain astrophysical parameters. In addition, in analyses of experimental data, it is typical to fit for the noise level as a free parameter. This serves to detect systematic errors and to improve the numerical stability of nested sampling for strongly peaked likelihoods. We will follow this experimental approach here applying it to our synthetic data. For the noise on the 21-cm global signal *σ*_gs_, we consider a log-uniform prior between 1 and 1,000 mK, and for the effective observation time for the 21-cm power spectrum *t*_obs_, we consider a log-uniform prior between 1 h and 10^5^ h of observation.

Alongside our priors to fully specify our Bayesian analysis, we need to establish our likelihood functions. We use a white Gaussian noise likelihood for our REACH analysis

10 $$\log ({\mathcal{L}}(D| \theta ))=\sum _{i}\left(-\frac{1}{2}\log (2\pi {\sigma }_{{\rm{gs}}}^{2})-\frac{1}{2}{\left(\frac{{T}_{{\rm{D}}}({\nu }_{i})-{T}_{21}(\theta ,{\nu }_{i})}{{\sigma }_{{\rm{gs}}}}\right)}^{2}\right),$$

where *ν*_*i*_ are the REACH measurement frequencies, *T*_D_ is the global 21-cm signal synthetic measurement data and *T*_21_ is the model of the global 21-cm signal. Meanwhile, for our SKA-Low analysis, we use an achromatic Gaussian likelihood

11 $$\begin{array}{l}\log ({\mathcal{L}}(D| \theta ))=\sum _{i}\sum _{j}\left(-\frac{1}{2}\log \left(2\pi {\sigma }_{i,\,j}{({t}_{{\rm{obs}}})}^{2}\right)\right.\\\left.-\displaystyle\frac{1}{2}{\left(\frac{{\varDelta }_{{\rm{D}}}^{2}({\nu }_{i},{k}_{j})-{\varDelta }_{21}^{2}(\theta ,{\nu }_{i},{k}_{j})}{{\sigma }_{i,\,j}({t}_{{\rm{obs}}})}\right)}^{2}\right),\end{array}$$

where *ν*_*i*_ are now the SKA-Low measurement frequencies, *k*_*j*_ is the SKA-Low measurement wavenumber, ${\varDelta }_{{\rm{D}}}^{2}$ is the 21-cm power spectrum synthetic measurement data, ${\varDelta }_{21}^{2}$ is the model of the 21-cm power spectrum and *σ*_*i*,*j*_(*t*_obs_) is the expected noise at the corresponding frequency and wavenumber after *t*_obs_ of observation. Finally, for our joint analyses, we assume the noise between the experiments is independent, so we combine the likelihoods multiplicatively.

## Supplementary information

Supplementary Information Supplementary Discussion, Tables 1 and 2, Figs. 1–5 and reference for Supplementary Discussion.

## Data Availability

The Pop III star Lyman-band spectra and specific X-ray emissivities used in this work are available via Zenodo at 10.5281/zenodo.5553052 (ref. ^99^) and 10.5281/zenodo.11502235 (ref. ^100^), respectively. All nested sampling chains resulting from our constraints forecasts are available via Zenodo at 10.5281/zenodo.11502235 (ref. ^101^). The intermediate data products created as part of this study are available upon reasonable request to the corresponding author.

## Code availability

The stellar evolution codes MESA and BINARY_C are available via GitHub at https://github.com/MESAHub/mesa/ and https://binary_c.gitlab.io/, respectively. TLUSTY, the stellar atmosphere code, is available at https://www.as.arizona.edu/~hubeny/pub/. GLOBALEMU and SCIKIT-LEARN, the codes we used to develop our emulators, are available via GitHub at https://github.com/htjb/globalemu and at https://scikit-learn.org/stable/install.html respectively. The nested sampler POLYCHORD is available via GitHub at https://github.com/PolyChord/PolyChordLite, and the nested sampling postprocessing tool ANESTHETIC^102^ is available via GitHub at https://github.com/handley-lab/anesthetic. 21CMSPACE and all scripts created as part of this study, such as plotting scripts, are available upon reasonable request to the corresponding author.
