# Supplementary material for: Determination of the mass distribution of the first stars from the 21-cm signal
Source: Nat Astron. 2025 Jun 20;9(8):1268–79. doi: 10.1038/s41550-025-02575-x (PMC12360958; doi:10.1038/s41550-025-02575-x)
Supplement: Supplementary file 1 — Supplementary Discussion, Tables 1 and 2, Figs. 1–5 and reference for Supplementary Discussion. [file 41550_2025_2575_MOESM1_ESM.pdf]

nature astronomy

Supplementary information https://doi.org/10.1038/s41550-025-02575-x

Determination of the mass distribution of the first stars from the 21-cm signal

In the format provided by the authors and unedited

SUPPLEMENTARY INFORMATION

A. Physical origins of 21-cm signal differences between IMFs

We have shown that the 21-cm signal varies with the Pop III IMF (see *Main Text* Figure 1). To understand the physical origins of these differences, we modified 21cmSPACE so that we can select which pieces of physics are modelled self-consistently from the IMF, showing in Figure 1 the increasing 21-cm signal deviation between IMFs as we gradually incorporate the impacts of the IMF. Note, for the depicted 21-cm signals, all other astrophysical parameters (e.g., Pop II and Pop III star formation efficiencies, X-ray emissivity of Pop II stars, and the recovery time between Pop III and Pop II star formation) are fixed at the values used to generate our synthetic measurement data, as listed in Table I. When we consider only the Pop III Lyman-band emissivity as IMF-dependent, the resultant variations in Wouthuysen-Field coupling, Ly α heating, and Lyman-Werner feedback, cause small $\Delta z < 1$ differences in the locations of the absorption trough and the high-redshift ($z \sim 20$) peak of the 21-cm power spectrum. These differences are enhanced to $\Delta z \sim 1$ when we account for the fact that Pop III star emission is not instantaneous but instead is spread over the finite lives of the stars (as previously discussed in [1]). The inclusion of this non-instantaneous emission has the most significant impact on the 21-cm signal calculated with a bottom-heavy IMF, as in these cases the mass-weighted mean stellar lifetime is larger than that of top-heavy IMFs, e.g., 1320 Myr for the *Sal* IMF versus only 2.2 Myr for the *Top* IMF. After the global 21-cm signal minimum ($z \approx 17$), we find the 21-cm signal converges between the considered IMFs due to the saturation of the Wouthuysen-Field coupling and Ly α heating being negligible compared to X-ray heating (which at this stage is IMF-independent). Hence, variations in the 21-cm signal due to the differences in Lyman-band emissivity of Pop III stars between IMFs are found to be isolated to high redshifts.

We now consider the impact of the IMF-dependent properties of Pop III XRBs on the 21-cm signal, which are new to this study. The variations in SED shape (not magnitude) introduce a small 2.6 mK spread in the depth of the 21-cm global signals and increase the differences between global signals at a fixed redshift in the $z < 18$ regime, with the largest difference being 4.8 mK at $z = 15$ (the $z = 15$ global signal spread was previously 1.5 mK without this effect). Similarly, enhanced divergences in the 21-cm power spectra are found at $z < 18$ that also peak around $z \sim 15$. These power spectra differences are larger at higher wavenumbers (3.4 and 16.7 mK^2 at $k = 0.2$ and 1.0 cMpc^{-1} respectively) though this is principally driven by the power spectrum increasing with k at these redshifts (the corresponding fractional differences are 13 % and 10 %).

Including the variation in the total X-ray emissivity with the Pop III IMF leads to much greater differences between 21-cm signals, producing: a 56 mK spread in the depth of the 21-cm global signals absorption trough, $\Delta z \sim 3$ differences in the timing of the 21-cm global signal minimum and the cosmic dawn peak in the power spectrum, and a secondary peak to appear in the 21-cm power spectra at $z \sim 17$ for some IMFs. This secondary power spectrum peak, often called the heating peak, occurs if heating in the early Universe is strong enough to dominate the fluctuations in the 21-cm signal [2]. As such, this peak is only seen for the IMFs with efficient X-ray emission (as seen in *Main Text* Figure 1). Furthermore, these more efficient X-ray emitting IMFs have higher and earlier emission peaks ($z \sim 12$) in the 21-cm global signal. To summarize, we find that, unlike the Lyman-band induced differences, the signatures of the Pop III IMF imprinted by X-rays are primarily at redshifts lower than $z = 23$ and are of greater magnitude.

Combined, the total impact of the Pop III IMF on the 21-cm signal produces large differences in both the 21-cm global signal and power spectrum from $z \sim 30$ down to $z \sim 8$. While the exact magnitude of these differences is dependent on other uncertain high-redshift astrophysical processes, it is clear from this example that the Pop III IMF can strongly affect the observable 21-cm signal. Additional differences in the 21-cm signal between Pop III IMFs may also be induced at lower redshifts due to the Pop III contribution to reionization . Top-heavy Pop III IMFs are anticipated to be more efficient at emitting ionizing photons than the bottom-heavy ones [3]. However, we do not include this effect in this work as the Pop III contribution to reionization is expected to be subdominant to that of Pop II stars, and hence, the differences in reionization history induced by varying the Pop III IMFs are anticipated to be small.

B. 21cmSPACE parameter priors and synthetic data values

In Table I, we list the astrophysical and cosmological parameters of the 21cmSPACE simulation code. Additionally, we provide the priors used for these parameters as part of our Bayesian analysis and the values used for these parameters in the generation of our synthetic data sets. The former are intentionally broad due to the large theoretical uncertainties in many of these parameters, whereas the latter are motivated by the cited works. Details of how these priors and synthetic data sets are used within our forecasting approach, as well as the motivation for this methodology, are discussed in the main text and *Methods* section.

C. 21-cm signal emulator performance metrics

In *Methods*, we outlined the architecture, training data, and overall performance of the neural network emulators we used in this study. Table II provides a detailed breakdown of the accuracy statistics of these twelve emulators. For global 21-cm signal emulators, we utilize the RMSE error metric, and for 21-cm power spectrum emulators, RMSMFE. We report for each emulator its mean, 68% and 95% error metrics over its testing set to demonstrate typical performance and the tail of the error distributions. Previous works developing 21-cm signal emulators, which included the ability to model excess radio backgrounds (as this work does), have achieved a 95% RMSE of 20.53 mK [4] for 21-cm global signal emulation and a typical RMSMFE of 20% [5] for 21-cm power spectrum emulation. While the accuracy we achieve depends on the IMF, in all cases, our emulator accuracies are comparable to, or better than, those stated in the literature.

D. IMF constraints from a joint analysis of REACH and SKA-Low

Alongside our forecasts for REACH and SKA-Low individually, we consider three scenarios for a joint analysis of data from these two experiments. In our pessimistic, moderate and optimistic scenarios, we combine 250, 25, and 5 mK sensitivity measurements by REACH with 300, 1000, and 3000 h of SKA-Low observations. We show the resulting forecast constraints in Figure 2 alongside those from REACH and SKA-Low individually. For this comparison, we present the prospective IMF constraints as Bayes ratios (the ratio of Bayesian evidences or, equivalently for our analysis, the ratio of posterior probabilities) between the data IMF and each alternative IMF to ensure that small changes in constraining power are visible.

The increase in the magnitude of the log Bayes ratios in the joint analyses compared to their composite individual experiments shows the disfavouring of all alternative IMFs is increased via a joint analysis. Hence, as discussed in the *Results* section, the joint analysis provides improved constraints compared to either of the experiments individually. In the pessimistic case, the joint analysis gives small benefits with all improvements in log Bayes ratio less than 2 compared to REACH at 250 mK on its own. Whereas the moderate and optimistic joint analysis cases show more benefit, with all log Bayes ratios improving by more than 1.5 (i.e., in all cases, the relative confidence in the data IMF versus an alternative IMF increases by > 4.48 times). For the combinations considered here, REACH appears to be the more constraining component of the joint analysis. However, this is likely caused by our intentionally conservative modelling of SKA-Low, with the real SKA-Low anticipated to provide significantly stronger constraints from a given amount of observation time than presented here. Regardless of which experiment is dominant in actuality, the improvements in constraints we see motivate the use of joint analysis of 21-cm global signal and 21-cm power spectrum data when constraining the Pop III IMF.

E. Robustness of conclusions to cosmic IMF

We previously presented the Pop III IMF constraints inferred from synthetic 21-cm signal measurements generated assuming the *Int-1* IMF. However, the actual Pop III IMF of the Universe is unknown. Hence, to show our main conclusions are robust within the uncertainties surrounding the Pop III IMF, we repeat our forecasts on synthetic measurements generated using each one of the six IMFs considered in this study. We depict the forecast IMF constraints from REACH at 25 mK sensitivity in Figure 3 and from SKA-Low for 3000 h of observation in Figure 4. For each synthetic measurement data set, the underlying 21-cm signal is simulated using the same set of high-redshift astrophysical parameters listed in Table I, and we use the same priors and analysis techniques as our forecasting analysis described in *Methods*.

For REACH at 25 mK sensitivity, we find the data IMF is always correctly identified as consistent with the synthetic measurement. Furthermore, at least two alternative IMFs are always disfavoured at $> 3\,\sigma$, and all alternative IMFs are disfavoured at $> 2\,\sigma$ with one exception (*Top* IMF when the *Sal* IMF is the data IMF). Our conclusion that a global 21-cm signal measurement at the expected 25 mK sensitivity of REACH will be able to provide $> 3\,\sigma$ significance constraints on the Pop III IMF is thus robust to the variations in the actual cosmic IMF.

Similarly, we find that 3000 h of SKA-Low observation can determine the Pop III IMF, regardless of the actual mass distribution of the first stars. The data IMF is always consistent with the mock measurement, and all other IMFs are disfavoured at $> 3\,\sigma$, with some ruled out at $> 5\,\sigma$. When the synthetic measurement data was generated assuming a *Int-0.5* or *Int-2* IMF, the constraints are particularly strong, with all alternative IMFs ruled out at $> 5\,\sigma$.

F. Biased Inference from a Mismatched Pop III IMF Assumption

The sensitivity of an observable (e.g. the 21-cm signal) to a parameter (e.g. the Pop III IMF) is a double-edged sword. It implies the potential to constrain the parameter but also that assuming an incorrect value for the parameter will likely bias estimation of other parameters from that data.

We show the values inferred for select high-redshift astrophysical parameters from our synthetic 25 mK and 5 mK sensitivity measurements of the 21-cm global signal by REACH in Figure 5. When our guess of the IMF is correct, and the model has the same IMF as was used to create the synthetic data (in this case, *Int-1*), we find the inferred parameter values to be consistent with the values used for data generation. However, we find biases when there is a mismatch between the data IMF and the IMF used in fitting (in this case, *Int-0*). For example, at 25 mK sensitivity, the parameter values that were used to generate the data (i.e., the true values) lie outside some 2D 95% confidence regions, with biases worsening to several sigma as sensitivity improves to 5 mK. We find biased parameter constraints to be a generic consequence of assuming an incorrect IMF, occurring regardless of the details of the data IMF, and in both REACH and SKA-Low inferences once sufficient sensitivity is reached. Thus, to ensure reliable inferences, future 21-cm signal data analyses must either fit for Pop III IMF or justify their results being robust to the uncertainties in the Pop III IMF.

The underlying cause of these biases is that the model can partially compensate for an incorrect IMF choice by adjusting the values of other parameters. However, as we saw in the *Results* section, the effect of the Pop III IMF is not fully degenerate with other parameters, and this compensation is thus only partial, leaving the 21-cm signal able to distinguish between Pop III IMFs.

I. SUPPLEMENTARY TABLES

Parameter	Explanation	Prior Type	Prior Range	Value used for Synthetic Data
V_c (km s^{-1})	Minimum halo virial circular velocity for star formation	Log Uniform	4.2 to 50	4.2 [6–8]
t_{delay} (Myr)	Recovery time between Pop III and Pop II star formation	Log Uniform	10 to 100	30 [9, 10]
$f_{*,II}$	Pop II star formation efficiency	Log Uniform	0.002 to 0.2	0.03 [11–13]
$f_{*,III}$	Pop III star formation efficiency	Log Uniform	0.001 to 0.1	0.005 [1, 12, 14]
$f_{X,II}$	X-ray emission efficiency of Pop II star forming haloes	Log Uniform	0.003 to 300	1 [15, 16]
f_{rad}	Radio emission efficiency of high-redshift galaxies	Log Uniform	0.3 to 30000	1 [17, 18]
ζ	Effective ionizing efficiency of high-redshift galaxies	Fixed	N/A	15 [19, 20]
R_{max} (cMpc)	Maximum mean free path of ionizing photons	Fixed	N/A	40 [21]
SED_{II}	Spectral energy distribution of Pop II XRBs	Fixed	N/A	Fragos et al. 2013 [15]

Supplementary Table I. **21cmSPACE physical parameters, their priors, and values used when generating our synthetic measurement data.** In addition to the parameters listed here, 21cmSPACE takes the Pop III IMF as an input parameter, from which $f_{X,III}$, SED_{III}, and the Lyman-band emissivity of Pop III star forming haloes are derived. By fitting for these parameters, we can explore how assuming an incorrect IMF will bias other inferences from 21-cm data and ensure any degeneracies between these parameters and the Pop III IMF, which will weaken IMF constraints, are accounted for in our forecasts. ζ, R_{max} (sometimes called R_{mfp} in previous works), and SED_{II} are fixed in this study as their impacts on the 21-cm signal are small or principally at low redshifts, where the 21-cm signal is largely insensitive to the Pop III IMF. To ensure realistic forecasts, the values used to generate the synthetic measurement data are, where available, taken to be consistent with current observations or theoretical predictions (see cited works).

IMF	21-cm global signal emulator error (mK)			21-cm power spectrum emulator error		
	68% RMSE	mean RMSE	95% RMSE	68% RMSMFE	mean RMSMFE	95% RMSMFE
Sal	6.97	8.09	25.71	0.07	0.07	0.13
Int-2	4.90	5.95	15.95	0.07	0.06	0.11
Int-1	4.92	6.14	18.04	0.06	0.06	0.10
Int-0.5	4.75	5.31	15.16	0.07	0.07	0.11
Int-0	2.94	3.27	8.96	0.06	0.06	0.09
Top	5.09	5.70	15.62	0.08	0.07	0.12

Supplementary Table II. **Accuracy of neural network emulators.** For each of our six IMF options, we train a separate emulator of the 21-cm global signal and 21-cm power spectrum, resulting in twelve emulators in total. We give the 68%, mean, and 95% percentile values of each emulator's error statistic across its testing set. The error on our global signal emulators is found to be long-tailed, with a small number of the 21-cm signals in our testing sets having RMSE > 10 mK. We find these higher error global signals all display very deep minima due to having a high astrophysical radio background [$f_{rad} \gg 1$, 18] which enhances the magnitude of the 21-cm signal by increasing T_γ. For our synthetic measurement data, we use a weak astrophysical radio background ($f_{rad} = 1$) that does not lead to any significant enhancement in the 21-cm signal magnitude [see *Main Text* Figure 1 and, 22]. Consequently, the regions of parameter space in which the emulator is less accurate are always strongly ruled out in our analyses (confirmed via our posteriors on f_{rad}). Hence, we do not anticipate this long tail in RMSE to impact our results.

II. SUPPLEMENTARY FIGURES WITH LEGENDS/CAPTIONS

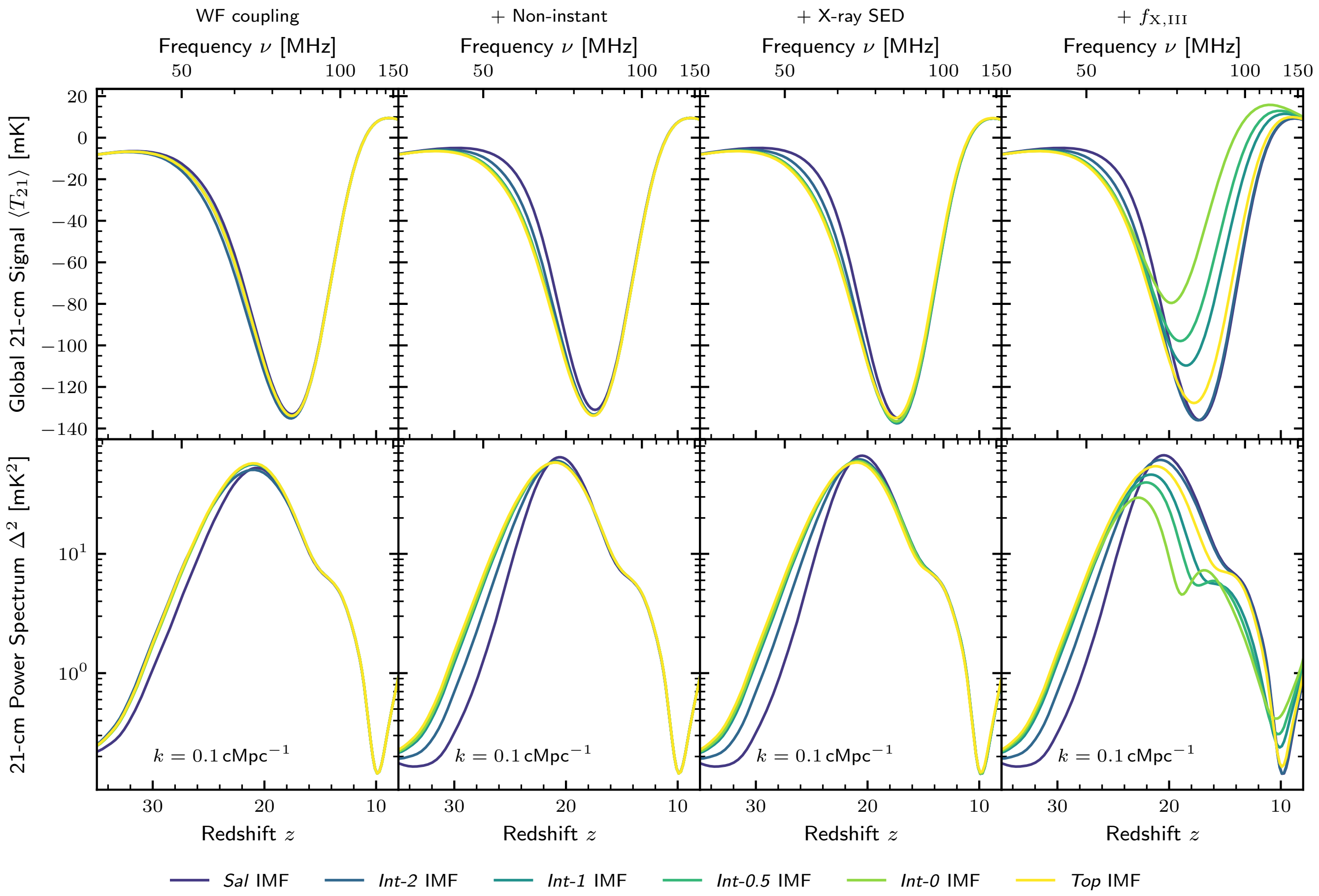

Supplementary Figure 1. **Increasing variation of the 21-cm signal between Pop III IMFs with progressive inclusion of IMF-dependent mechanisms.** We show the 21-cm global signal (top) and $k = 0.1$ cMpc^{-1} 21-cm power spectrum (bottom) for our six example Pop III IMFs. From left to right, we progressively integrate the dependence on the IMF, starting with the Lyman-band emission of the Pop III stars (WF coupling column), then the Pop III star lifetimes (Non-instant column), Pop III XRB SED shape (X-ray SED column), and Pop III XRB total X-ray emissivity ($f_{\mathrm{X,III}}$ column). Thus, the rightmost column represents self-consistent modelling of the impacts of the Pop III IMF on the 21-cm signal and matches the signals shown in *Main Text* Figure 1. At $z < 23$, we find that differences in the 21-cm signal between IMFs are principally driven by Pop III XRB emissivity. Conversely, a combination of Lyman-band emissivity and stellar lifetime causes higher redshift variations between IMFs.

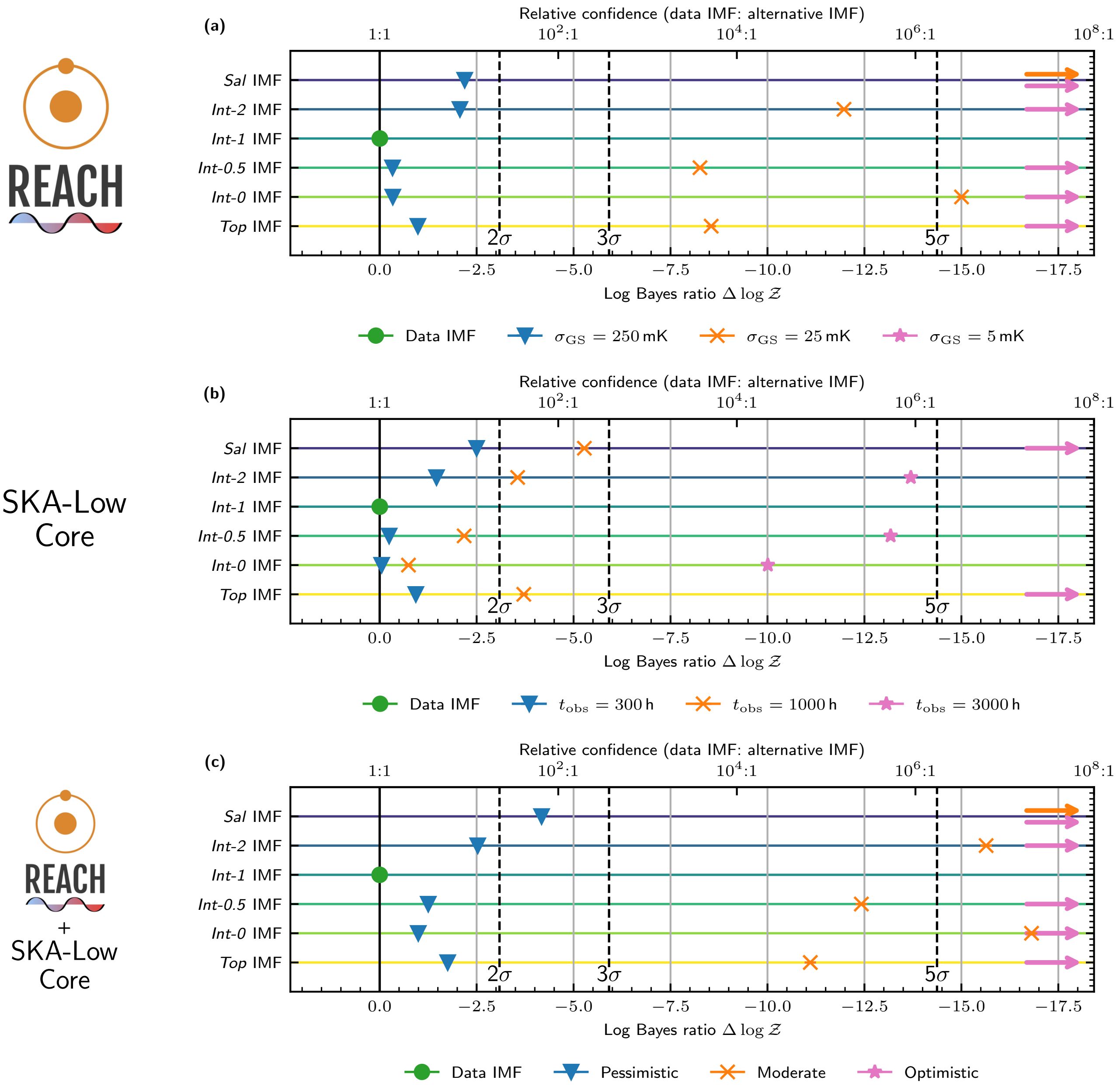

Supplementary Figure 2. **Comparison of the prospective constraints on the Pop III IMF from individual experiments and joint analyses.** Each panel shows the prospective constraints on the Pop III IMF from mock 21-cm signal data analyses, expressed as the logarithm of the Bayes ratio between the fit with the data IMF (*Int-1*) and the fit with an alternative IMF (top axes show the equivalent relative confidence, or betting odds, between the data IMF and the alternative IMF). For convenience, when the alternative IMF is rejected at 2, 3, and 5 σ in this pairwise comparison is shown as vertical dashed lines. We show the REACH, SKA-Low, and joint analysis constraints in the top (**a**), middle (**b**), and bottom (**c**) rows at different sensitivities indicated by colour. The joint analysis improves the IMF constraints over either of the individual experiments in all cases, motivating the use of joint 21-cm data analyses in future attempts to constrain the Pop III IMF.

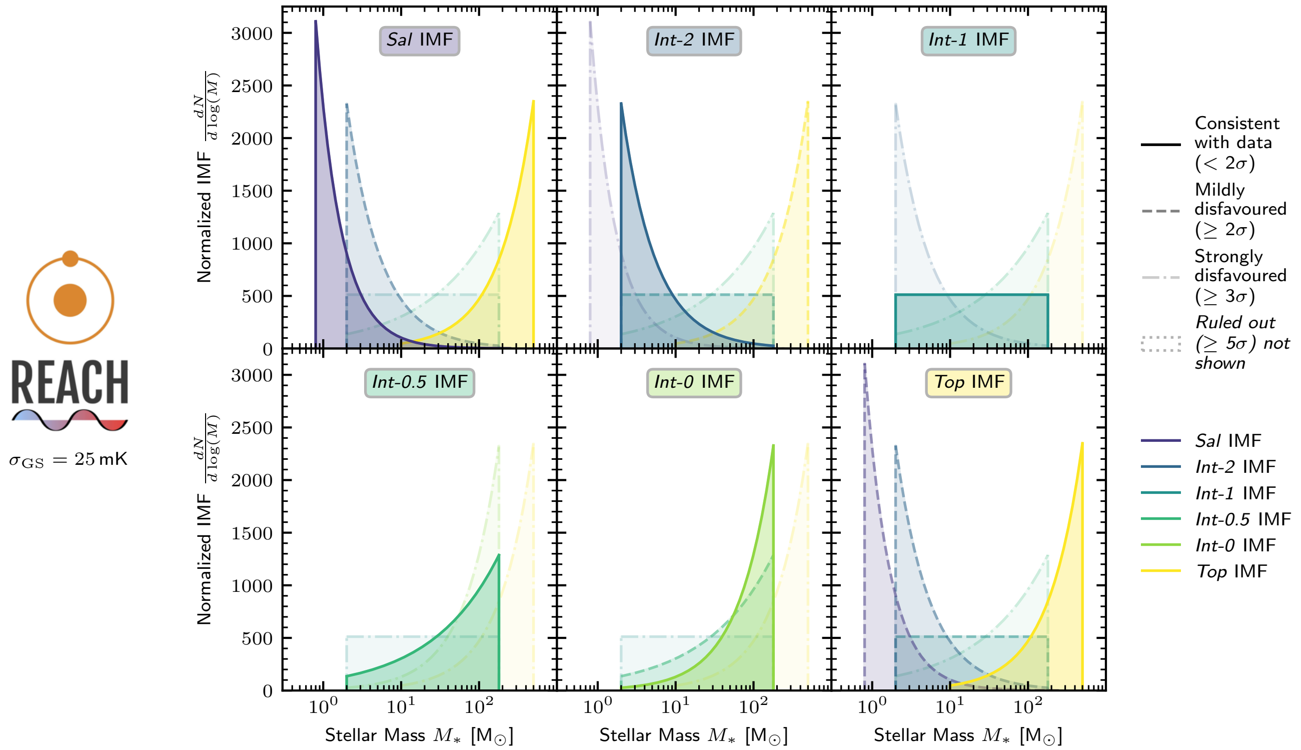

Supplementary Figure 3. **Prospective constraints on the mass distribution of the first stars from REACH.** As in *Main Text* Figure 2, each panel shows the posterior confidence in the six Pop III IMFs considered in this study, indicated via line type and opacity. However, all constraints here are from REACH at 25 mK sensitivity, with the different panels showing the constraints when different Pop III IMFs are used to generate the synthetic measurement data (as indicated by panel labels). For all six cases, we find the data IMF is the most likely, and multiple alternative IMFs are disfavoured at $> 3\,\sigma$. Furthermore, all alternative IMFs are disfavoured at $> 2\,\sigma$, except for the *Top* IMF when *Sal* IMF is used to generate the data. Therefore, we demonstrate that a global 21-cm signal experiment, such as REACH, should be able to constrain the Pop III IMF regardless of the true cosmic Pop III IMF.

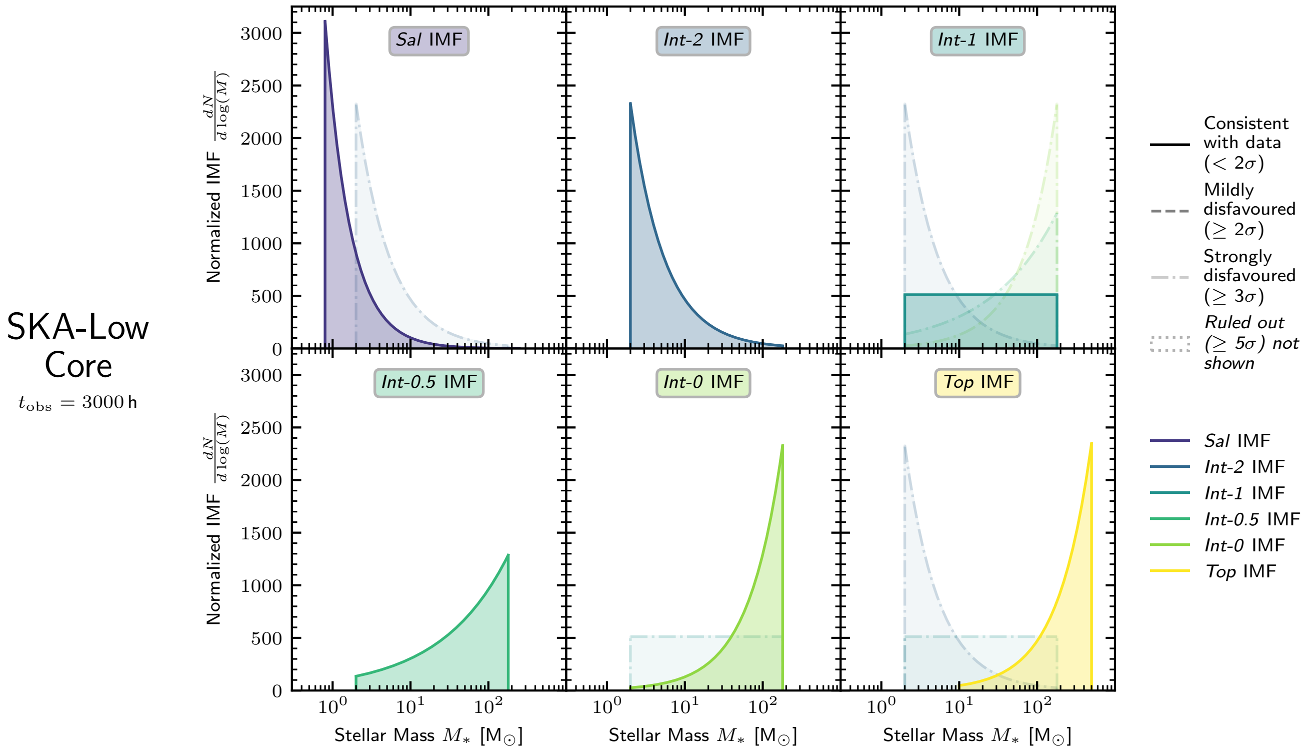

Supplementary Figure 4. **Prospective constraints on the mass distribution of the first stars from SKA-Low.** As in *Main Text* Figure 2, each panel shows the posterior confidence in the six Pop III IMFs considered in this study, indicated via line type and opacity. However, all constraints here are from 3000 h of foreground-avoidance observations by SKA-Low, with the different panels showing the constraints when different Pop III IMFs are used to generate the synthetic measurement data (as indicated by panel labels). For all six cases, we find the data IMF is the most likely, with all alternative IMFs disfavoured at $> 3\,\sigma$ and multiple alternative IMFs ruled out at $> 5\,\sigma$. We thus find that strong constraints on the Pop III IMF are expected from SKA-Low after 3000 h of observation, irrespective of the true cosmic Pop III IMF.

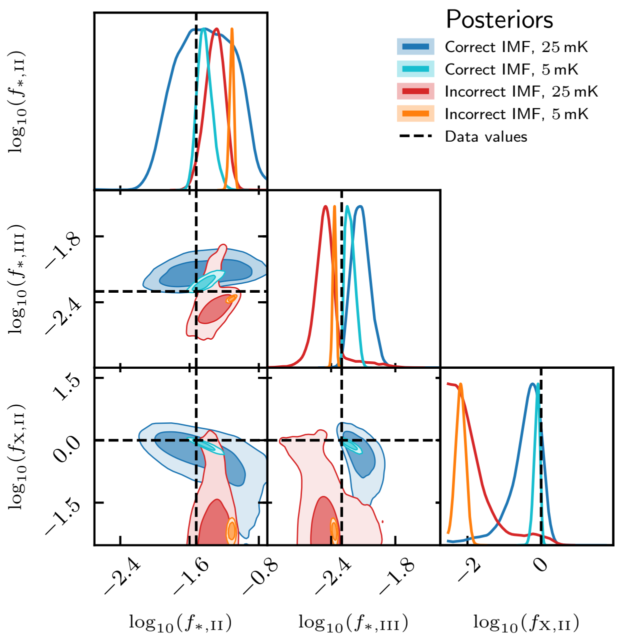

Supplementary Figure 5. **Biased parameter constraints from 21-cm global signal observations when assuming an incorrect IMF.** Dark and light regions show 1 and 2 σ credible regions respectively. The 21-cm signal model used in the synthetic data for this figure assumed an *Int-1* IMF, a Pop II star formation efficiency of $f_{*,\mathrm{II}} = 0.03$, a Pop III star formation efficiency of $f_{*,\mathrm{III}} = 0.005$, and a Pop II X-ray relative emissivity of $f_{\mathrm{X}} = 1$ (black dashed lines). When fitting this synthetic data with a model that assumes the correct IMF parameter inferences are unbiased (blue and cyan regions). Conversely, significant parameter biases are seen when the synthetic data is fit with a model assuming an incorrect IMF (in this case, *Int-0*). Similar biases occur in other 21cmSPACE parameters and when the other IMFs from *Main Text* Table 1 are erroneously assumed to be true. Note that these parameter posteriors are non-Gaussian, retroactively providing additional justification for using a nested-sampling method over Fisher forecasts [23] for our analysis.

III. REFERENCES

---

[1] Gessey-Jones, T. *et al.* Impact of the primordial stellar initial mass function on the 21-cm signal. *Mon. Not. R. Astron. Soc.* **516**, 841–860 (2022).

[2] Barkana, R. The rise of the first stars: Supersonic streaming, radiative feedback, and 21-cm cosmology. *Phys. Rep.* **645**, 1–59 (2016).

[3] Schaerer, D. On the properties of massive Population III stars and metal-free stellar populations. *Astron. Astrophys.* **382**, 28–42 (2002).

[4] Bevins, H. T. J. *et al.* Joint analysis constraints on the physics of the first galaxies with low-frequency radio astronomy data. *Mon. Not. R. Astron. Soc.* **527**, 813–827 (2024).

[5] Abdurashidova, Z. *et al.* HERA Phase I Limits on the Cosmic 21 cm Signal: Constraints on Astrophysics and Cosmology during the Epoch of Reionization. *Astrophys. J.* **924**, 51 (2022).

[6] Fialkov, A., Barkana, R., Tseliakhovich, D. & Hirata, C. M. Impact of the relative motion between the dark matter and baryons on the first stars: semi-analytical modelling. *Mon. Not. R. Astron. Soc.* **424**, 1335–1345 (2012).

[7] Schauer, A. T. P., Glover, S. C. O., Klessen, R. S. & Clark, P. The influence of streaming velocities and Lyman-Werner radiation on the formation of the first stars. *Mon. Not. R. Astron. Soc.* **507**, 1775–1787 (2021).

[8] Gessey-Jones, T., Fialkov, A., de Lera Acedo, E., Handley, W. J. & Barkana, R. Signatures of cosmic ray heating in 21-cm observables. *Mon. Not. R. Astron. Soc.* **526**, 4262–4284 (2023).

[9] Chiaki, G., Susa, H. & Hirano, S. Metal-poor star formation triggered by the feedback effects from Pop III stars. *Mon. Not. R. Astron. Soc.* **475**, 4378–4395 (2018).

[10] Magg, M. *et al.* Effect of the cosmological transition to metal-enriched star formation on the hydrogen 21-cm signal. *Mon. Not. R. Astron. Soc.* **514**, 4433–4449 (2022).

[11] Mirocha, J., Furlanetto, S. R. & Sun, G. The global 21-cm signal in the context of the high- z galaxy luminosity function. *Mon. Not. R. Astron. Soc.* **464**, 1365–1379 (2017).

[12] Muñoz, J. B. *et al.* The impact of the first galaxies on cosmic dawn and reionization. *Mon. Not. R. Astron. Soc.* **511**, 3657–3681 (2022).

[13] Pochinda, S. *et al.* Constraining the properties of Population III galaxies with multiwavelength observations. *Mon. Not. R. Astron. Soc.* **531**, 1113–1132 (2024).

[14] Gurian, J., Jeong, D. & Liu, B. Zero Metallicity with Zero CPU Hours: Masses of the First Stars on the Laptop. *Astrophys. J.* **963**, 33 (2024).

[15] Fragos, T., Lehmer, B. D., Naoz, S., Zezas, A. & Basu-Zych, A. Energy Feedback from X-Ray Binaries in the Early Universe. *Astrophys. J. Lett.* **776**, L31 (2013).

[16] Fialkov, A., Barkana, R. & Visbal, E. The observable signature of late heating of the Universe during cosmic reionization. *Nature* **506**, 197–199 (2014).

[17] Gürkan, G. *et al.* LOFAR/H-ATLAS: the low-frequency radio luminosity-star formation rate relation. *Mon. Not. R. Astron. Soc.* **475**, 3010–3028 (2018).

[18] Reis, I., Fialkov, A. & Barkana, R. High-redshift radio galaxies: a potential new source of 21-cm fluctuations. *Mon. Not. R. Astron. Soc.* **499**, 5993–6008 (2020).

[19] Furlanetto, S. R., Oh, S. P. & Briggs, F. H. Cosmology at low frequencies: The 21 cm transition and the high-redshift Universe. *Phys. Rep.* **433**, 181–301 (2006).

[20] Planck Collaboration *et al.* Planck 2018 results. VI. Cosmological parameters. *Astron. Astrophys.* **641**, A6 (2020).

[21] Wyithe, J. S. B. & Loeb, A. A characteristic size of 10 Mpc for the ionized bubbles at the end of cosmic reionization. *Nature* **432**, 194–196 (2004).

[22] Sikder, S., Barkana, R., Fialkov, A. & Reis, I. Strong 21-cm fluctuations and anisotropy due to the line-of-sight effect of radio galaxies at cosmic dawn. *Mon. Not. R. Astron. Soc.* **527**, 10975–10985 (2024).

[23] Fisher, R. A. On the Mathematical Foundations of Theoretical Statistics. *Philosophical Transactions of the Royal Society of London Series A* **222**, 309–368 (1922).
